# Free flap reconstruction of Achilles tendon and overlying skin defect using ALT and TFL fabricated chimeric flap

**DOI:** 10.1080/23320885.2019.1635023

**Published:** 2019-07-04

**Authors:** Junji Ando, Minoru Sakuraba, Atsushi Sugawara, Aya Goto, Shuchi Azuma, Nobuyuki Mitsuhashi, Kousuke Sasaki, Itaru Sone

**Affiliations:** a Department of Plastic and Reconstructive Surgery, Iwate Medical University Hospital, Morioka, Japan;; b Department of Orthopedic Surgery, Iwate Medical University Hospital, Morioka, Japan

**Keywords:** Chimeric free flap, Achilles tendon, reconstruction, anterolateral thigh flap, tensor fascia latae flap

## Abstract

A 33-year-old man developed a left Achilles tendon rupture and skin necrosis. We reconstructed the defect using an anterolateral thigh flap and a tensor fasciae lata muscle flap in a chimeric fashion. he was able to stand on a toe of the operated foot without help 6 months postoperatively.

## Introduction

A combined defect of the Achilles tendon and the overlying skin may result from direct trauma or infection after the repair of a ruptured tendon.

It should be reconstructed with a strong tendon substitute and thin pliable skin to achieve satisfactory ambulatory function. Although an avascular fascia graft and local skin flap can be used to correct small defects of the Achilles tendon and overlying skin, the use of vascularised fascia and soft tissue is recommended in patients with large defects in the calcaneal region. Here we describe our experience with successful reconstruction of a complex Achilles tendon defect using a fabricated chimeric flap.

## Case report

**Figure 1.**
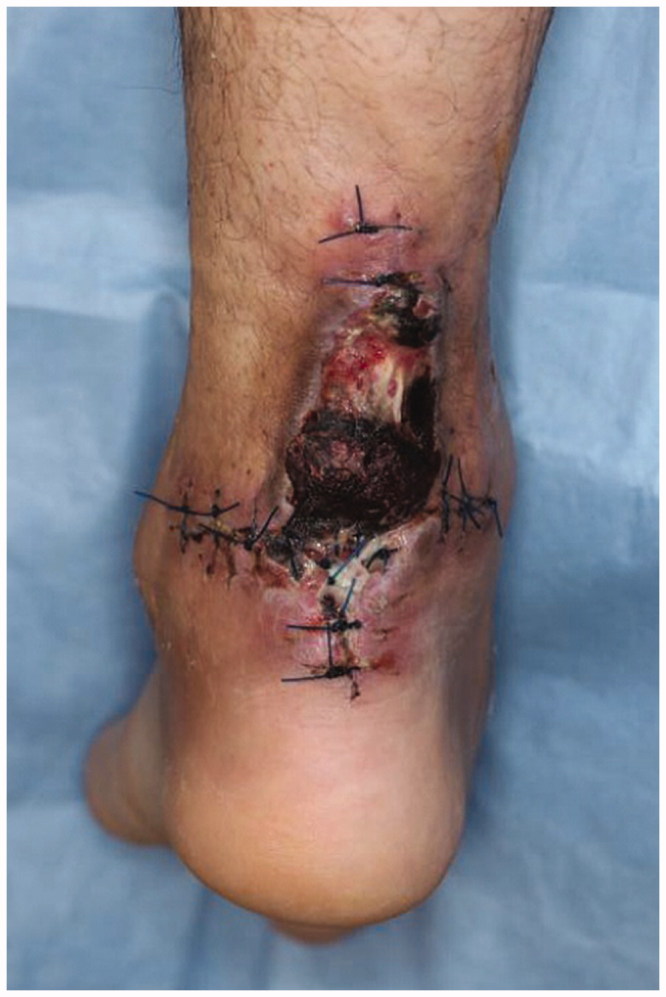
Photograph of the first patient visit 39 days after initial injury. Achilles tendon was re-ruptured with skin necrosis around it.

A 33-year-old man ruptured his left Achilles tendon while playing soccer and underwent a primary tendon repair in June 2017. One month after the primary repair, the Achilles tendon re-ruptured and re-anastomosis of the tendon was performed. However, the procedure failed due to local infection and skin necrosis. He ultimately presented to our department for reconstructive surgery using a free flap 39 days after the initial injury (Figure 1). Staphylococcus aureus were identified in bacterial culture results, patients were given by drip infusion 1g of cefazolin twice a day for after hospitalisation until 3 days after surgery.

We planned simultaneous reconstruction of the tendon and soft tissue defects using anterolateral thigh (ALT) and tensor fasciae latae (TFL) muscle chimeric flap using the lateral circumflex femoral system, skin coverage by an ALT flap, and Achilles tendon reconstruction using a TFL flap.

**Figure 2.**
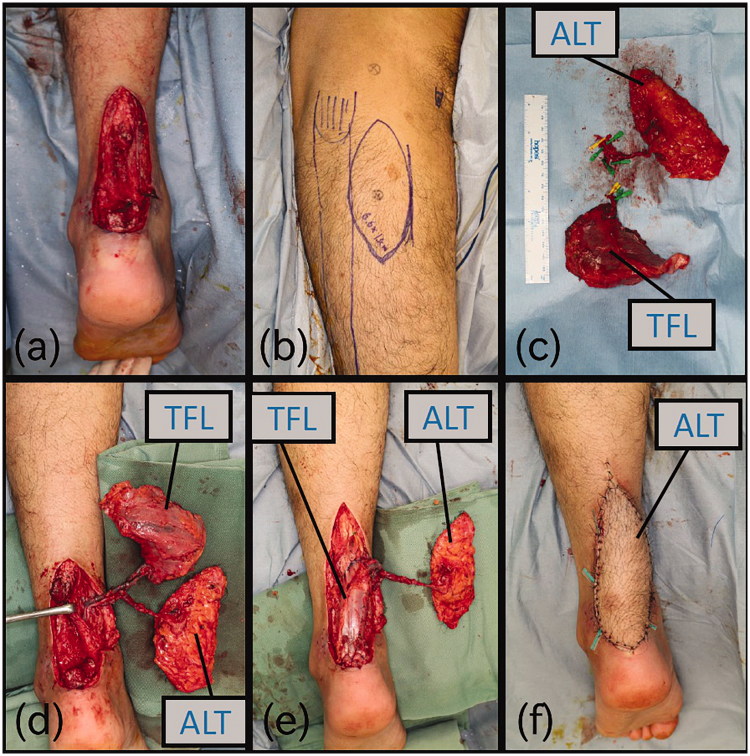
Intraoperative photographs (a) After debridement of soft tissue and the Achilles tendon. (b) Design of the flap. (c) TFL was sutured with Achilles tendon. (d) After the operation.

Surgical repair was performed 55 days after the primary rupture. The cutaneous perforator of the ALT flap was identified and marked on the skin preoperatively with a colour Doppler ultrasonography before the operation. Wide debridement resulted in a large combined Achilles tendon (7 cm long) and overlying skin defect (5.5 × 11 cm) (Figure 2(a)). The ALT flap with a 6.5 × 13 cm skin island without fascia and TFL musculofascial flap with an 11-cm length of iliotibial fascia were elevated from the right thigh (Figure 2(b)).

In this case, the descending branch of the lateral circumflex femoral artery (LCFA), the pedicle of the ALT flap, branched directly from the deep femoral artery or common femoral artery. As a result, the vascular pedicles of the ALT and TFL flaps were independent of each other. Therefore, the descending branch of the LCFA was anastomosed with the posterior tibialis vessels in an end-to-side fashion first, while the vascular pedicle of the TFL flap was anastomosed with the side branch of the descending branch of the LCFA in an end-to-end fashion.

After blood circulation was confirmed, the Achilles tendon reconstruction was performed by orthopaedic surgeons using Kirchmayer’s suture technique (Figure 2(c)).

Finally, the skin defect was covered with the ALT skin paddle (Figure 2(d)).

**Figure 3.**
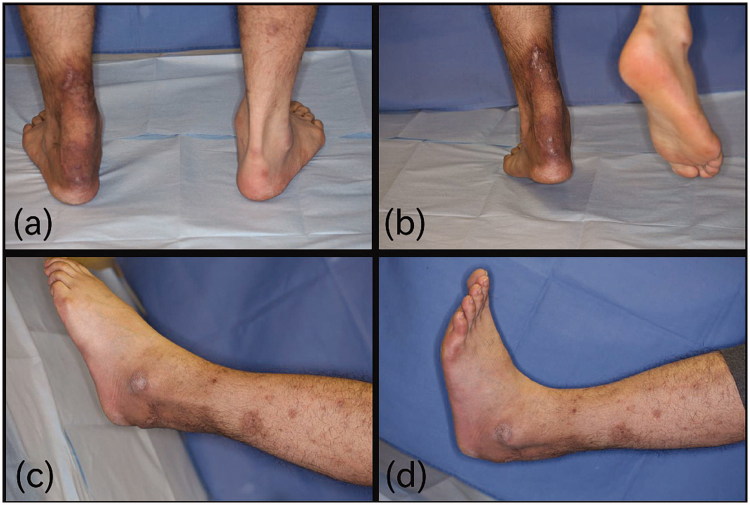
Six months after the operation. (a) He could stand on a toe of operated foot. (b) plantar flexion was 45 degree. (c) Dorsiflexion was 10 degree.

Ambulatory loading was commenced 3 weeks after surgery, and the patient could walk on his foot after 81 days of wearing an ankle brace. Six months postoperatively, he could stand on a toe of the operated foot without help (Figure 3(a,b)). Function at the ankle was good (Figure 3(c,d) and he had no disability in his daily life. He was aesthetically satisfied with the results.

## Discussion

Small defects of the Achilles tendon in the absence of soft-tissue defects can usually be repaired using transposition skin flap and avascular tendon transplantation. However, vascularised free flap reconstruction is recommended in patients with large skin defects or chronic wound infections. [1]

There are various Achilles tendon repair methods. The free inguinal flap with external oblique muscle fascia [2], free inferior glutaeal artery perforator flap with glutaeus maximus fascia [3], lateral arm flap with biceps brachialis muscle fascia [4], free dorsalis pedis flap extensor tendon [5], free lateral thigh flap with fascia lata [6], free TFL flap [7], and free ALT flap with fascia lata [8,9] were reported previously. A TFL flap contains enough iliotibial thick fascia to support weight at the Achilles tendon repair site. An ALT flap provides certain coverage of wide defects with thin pliable skin to support shoe wearing. We believe that combination of these two flaps can be the first choice for the reconstruction of complex Achilles tendon defects. However, other flaps can be used if the TFL or ALT flap is impossible to use because of prior injury. In this case, the skin defect was large and accompanied by an infection, so we used an ALT flap to cover the skin and used a TFL flap to reconstruct the Achilles tendon.

Compared to the reported cases, this chimeric fashion flap transfer is resistant to infection because the fascia lata is well vascularised. Furthermore, this chimeric flap transfer can provide freestyle flap repair between a tendon repairing flap and a skin repairing flap. The disadvantage is that the surgical procedure is somewhat complicated and difficult because of multiple vascular anastomosis.

In this case, the descending branch of the LCFA and pedicle of the TFL flap are uncommon, it was necessary to perform micro-anastomosis to create a fabricated chimeric flap. Lakhiani et al described that the descending branch of the LCFA arises from the deep femoral artery (6–13%) or from the common femoral artery (1–6%) [10]; therefore, we must take notice of the anatomy of the LCFA system to use this chimeric flap.
